# Correction: Alves et al. WNK2 Inhibits Autophagic Flux in Human Glioblastoma Cell Line. *Cells* 2020, *9*, 485

**DOI:** 10.3390/cells13141219

**Published:** 2024-07-19

**Authors:** Ana Laura Vieira Alves, Angela Margarida Costa, Olga Martinho, Vinicius Duval da Silva, Peter Jordan, Viviane Aline Oliveira Silva, Rui Manuel Reis

**Affiliations:** 1 Molecular Oncology Research Center, Barretos Cancer Hospital, 14784 400 Barretos, Brazil; alves.anav@gmail.com (A.L.V.A.); olgamartinho@med.uminho.pt (O.M.); vinids@gmail.com (V.D.d.S.); vivianeaos@gmail.com (V.A.O.S.); 2 Life and Health Sciences Research Institute (ICVS), School of Medicine, University of Minho, 4710-057 Braga, Portugal; angela.amorimcosta@ineb.up.pt; 3 ICVS/3B’s—PT—Government Associate Laboratory, 4806-909 Braga, Portugal; 4 Department of Human Genetics, National Health Institute Doutor Ricardo Jorge, 1649-016 Lisbon, Portugal; peter.jordan@insa.min-saude.pt; 5 BioISI—Biosystems & Integrative Sciences Institute, Faculty of Sciences, University of Lisbon, 1749-016 Lisbon, Portugal

**Error in Figure**

In the original publication [1], there was a mistake in Figure 2. The authors incorrectly repeated the same sequence of p-p70S6K protein during the assembly of the panel of Figure 2A. However, the new set of bands representative of the p-p70S6K protein does not change the results because these data are representative of two independent experiments. The corrected Figure 2 appears below. The authors state that the scientific conclusions are unaffected. This correction was approved by the Academic Editor. The original publication has also been updated. 

**Figure 2:**
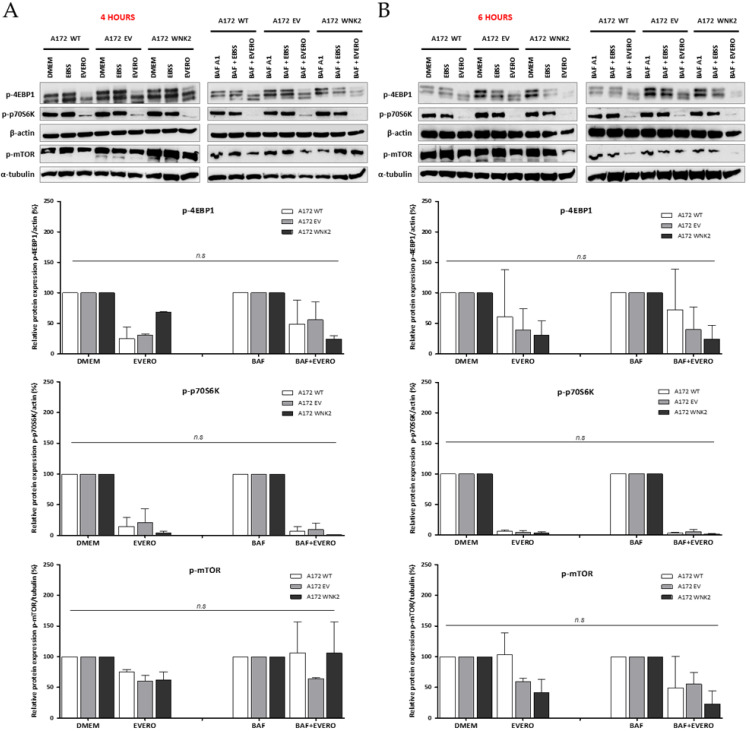
Evaluation of proteins involved in the mammalian target of rapamycin (mTOR) pathway by western blot. A172 WT, A172 EV, and A172 WNK2 cell lines were treated with bafilomycin A1 (BAF, 20 nM), starvation (EBSS medium), or everolimus (EVERO, 10 nM) for 4 (**A**) and 6 h (**B**). The protein extract was evaluated for phosphorylation of mTOR and its substrates p-p70S6K and p-4EBP1 by western blot. Normalized densitometric band intensities of mTOR activity used α-tubulin as an endogenous loading control. For the substrates p-p70S6K and p-4EBP1, β-actin was used as an endogenous control. The graphs are representative of two independent biological experiments. n.s.: Not significant.
